# Trends in feed evaluation for poultry with emphasis on in vitro techniques

**DOI:** 10.1016/j.aninu.2020.08.006

**Published:** 2021-01-09

**Authors:** Faegheh Zaefarian, Aaron J. Cowieson, Katrine Pontoppidan, M. Reza Abdollahi, Velmurugu Ravindran

**Affiliations:** a Monogastric Research Centre, School of Agriculture and Environment, Massey University, Palmerston North 4442, New Zealand; b DSM Nutritional Products, Wurmisweg 576, Kaiseraugst, Switzerland; c Novozymes A/S, DK-2800 Kgs, Lyngby, Denmark

**Keywords:** Poultry, Feed evaluation, In vivo assay, In vitro techniques, Protein, Energy

## Abstract

Accurate knowledge of the actual nutritional value of individual feed ingredients and complete diets is critical for efficient and sustainable animal production. For this reason, feed evaluation has always been in the forefront of nutritional research. Feed evaluation for poultry involves several approaches that include chemical analysis, table values, prediction equations, near-infrared reflectance spectroscopy, in vivo data and in vitro digestion techniques. Among these, the use of animals (in vivo) is the most valuable to gain information on nutrient utilization and is more predictive of bird performance. However, in vivo methods are expensive, laborious and time-consuming. It is therefore important to establish in vitro methods that are reliable, rapid and practical to assess the nutritional quality of feed ingredients or complete diets. Accuracy of the technique is crucial, as poor prediction will have a negative impact on bird performance and, increase feed cost and environmental issues. In this review, the relevance and importance of feed evaluation in poultry nutrition will be highlighted and the various approaches to evaluate the feed value of feed ingredients or complete diets will be discussed. Trends in and practical limitations encountered in feed evaluation science, with emphasis on in vitro digestion techniques, will be discussed.

## 1 Introduction

Accurate ingredient evaluation is central to precise and cost-effective feed formulations. Ingredient variability is inherent and unavoidable. The primary aim of feed evaluation is to provide the nutritionists with reliable data on the digestible nutrient and metabolizable energy contents of different ingredients, so that the expected variation between batches of ingredients could be incorporated into formulation matrices. It is well accepted that poultry production will be the fastest growing livestock sector of the future. This predicted growth will come with multiple challenges; the most profound effect will be on the appetite for raw materials. Since the global feed resource base is limited, it is evident that the future demand and supply of ingredients will always be tight (Abdollahi and Ravindran, 2019). In this context, the evaluation and use of alternative raw materials is an important strategy for the industry to expand in the future. When using such poorly digested alternative ingredients, feed formulation based on digestible nutrients is a requisite and, routine and better feed evaluation becomes even more pertinent.

During the past few decades, researchers have strived to develop better evaluation techniques, giving a clearer picture and understanding of how to improve poultry diets. Accurate evaluation will enable more precise formulations and will address most, if not all, of the future expectations in poultry nutrition including productivity, bird efficiency, environmental concerns, sustainability, gut health and profitability. In the commercial world, the matrix values used in poultry feed formulations are based on a combination of several sources: chemical analysis, table values, prediction equations, near-infrared reflectance spectroscopy (NIRS), actual animal research (in vivo) data and, in the case of some ingredients, in vitro tests (Leeson, 1997; Hughes and Choct, 1999). Accuracy of the source is the critical factor and imprecise matrices can result in higher feed cost, poor bird performance and an increased nutrient excretion into the environment. Among these sources, in vivo data provides the best information on the utilization of nutrients and are more predictive of bird performance. However, estimating the ingredient value by in vivo research is costly, laborious and time-consuming. It can be carried out only in established research institutions with special animal facilities, dedicated laboratories and trained technicians; most feed manufacturing companies lack these capabilities. For these reasons, the development of in vitro evaluations that are reliable, rapid and practical has attracted considerable attention over the years. Despite some fundamental differences between the digestion in pigs and poultry, almost all current in vitro poultry models are based on pigs (Boisen and Fernández, 1995, Boisen and Fernández, 1997; Regmi et al., 2008, 2009; Woyengo et al., 2015) and used for the generation of in vitro data for poultry (Losada et al., 2009, 2010; Palić et al., 2012).

In the main, feed evaluation science is focussed primarily on the assessment of energy and protein, the major quantitative and most costly components in feed formulations. Energy is the obvious first step in feed evaluation because of its importance in feed intake control, which drives bird growth, and diet cost. Animal growth is essentially made of proteins and hence protein digestibility assay is the second step in feed evaluation. In recent years, due to environmental concerns, there is also increasing interest in the measurement of utilization of phosphorus, the third costliest nutrient in poultry feed (Mutucumarana et al., 2014, 2015), and of calcium because of its close metabolic relationship with phosphorus (Anwar and Ravindran, 2016; David et al., 2019). Discussion of the utilization of these minerals is beyond the scope of the current review and, the focus will be on energy and protein evaluation.

The present paper covers an overview of the current trends in and various approaches to evaluate the nutritional value of feed ingredients and complete diets for poultry. Owing to the similarities in digestion between pigs and poultry, supporting data from pig research will be presented when needed. The emphasis of the review will be on in vitro digestion methodology, including key aspects to be considered and limitations to the successful development of suitable models.

## 2 Feed evaluation methods

### 2.1 Physical measurements

#### 2.1.1 Grain density and 1,000 kernel weight

Physical measurements of cereal grains such as density or 1,000-kernel weight have been historically used to describe feed quality (Sibbald, 1975; Sibbald and Price, 1976). This approach is faster and less expensive compared to chemical analyses. However, the consensus is that these methods are not good predictors of nutrient content or apparent metabolizable energy (AME) and, are inferior to predictions based on chemical measurements (Fairbairn et al., 1999; Zijlstra et al., 1999). Number of studies have investigated the relationship between physical characteristics and nutritive value of feed ingredients, with conflicting findings. For example, Wiseman and McNab (1995) reported a strong positive correlation between the AME and 1,000-kernel weight of wheat. In contrast, Hughes et al. (1996) and Garnsworthy et al. (2000) found that 1,000-kernel weight was not correlated with the AME of wheat. Similarly, Wiseman (2000) reported that there were no significant correlations between bushel weight and 1,000-kernel weight versus the AME value of 50 wheat samples (from 10 varieties) fed to broiler chickens. In a study by Pirgozliev et al. (2003), 1,000-kernel weight of wheat was negatively correlated (*r* = −0.564) with feed conversion efficiency in broiler chickens. Ball et al. (2013), on the other hand, reported a positive correlation between 1,000-kernel weight and dry matter (DM) intake (*r =* 0.30), weight gain (*r =* 0.34) and feed efficiency (*r =* 0.37), but correlation between 1,000-kernel weight and AME was not significant.

#### 2.1.2 Endosperm hardness

Hardness of the endosperm is an important characteristic in the quality of grains (Ball et al., 2013). Positive correlations between wheat endosperm hardness and growth performance have been reported in broilers (Scott et al., 1998; Pirgozliev et al., 2003). During the milling process, hard wheat breaks down and produce fine flour with regular particle sizes and large surface areas. Starch granules are often mechanically damaged during the hard wheat milling process. But, in soft wheat, milling produces flour with irregular particle sizes which has lower surface areas and lower starch damage (Ball et al., 2013). It has been suggested by Pirgozliev et al. (2003) that starch granule damage may be an important characteristic of wheat affecting the performance of birds. Nevertheless, Garnsworthy et al. (2000) found that the grain hardness showed no correlation with the nutritive value of wheat.

Overall, physical traits are not generally considered to be good predictors of nutritional quality of feed ingredients. However, some physical traits such as grain test weight is still used as a grain quality indicator and to set the prices. Buyers prefer higher test weight grains since the grain has a greater proportion of starch-rich endosperm and less bran and hull, and therefore more energy.

### 2.2 Chemical analyses

Characterization of feed ingredients in terms of gross content of nutrients and energy by chemical analysis is relatively simple and straight-forward. Proximate analysis is the widely and routinely used method to measure the nutrient content of feed ingredients and diets. This system, devised in the mid-nineteenth century at the Weende Experiment Station in Germany (Damodaran et al., 2008), provides crude measures of water, ash, fat, protein and fiber. Nitrogen-free extract (NFE), representing sugars and starches, is calculated by difference rather than measured by analysis. This system was developed at a time when the chemistry of food constituents was only partially understood and, the growth of nutritional sciences has shown that for nutritional studies a more detailed and biochemically oriented approach to food analysis is needed. Nevertheless, proximate analysis, including the original methods, still forms the first step in feed analysis. In recent decades, the terminology to describe crude fiber has become more refined with the advances in detergent fiber system (van Soest, 1963) and polysaccharide analysis (Nielsen, 2010).

Over the past 50 years, a great deal of research has been devoted to the development and improvement of prediction equations based on chemical analysis (Carpenter and Clegg, 1956; Fisher, 1982; Carré et al., 2013). However, major limitations in the use of routine chemical analysis are reproducibility of the chemical measurements, time required for the analyses, cost, the need for specific equipment for laboratory procedures and the use and disposal of hazardous chemicals used (Leeson, 1997; Noblet and Jaguelin-Peyraud, 2007). In general, chemical analyses only provide information on the nutrient content of feed ingredients or diets and are not able to take into consideration of the digestibility of nutrients. However, no common feed ingredient is 100% digested and their potential nutritive value is not fully realized at the animal level. The digestion of most substrates is incomplete, with 10% to 20% being normally undigested and excreted (Ravindran, 2013). The gross nutrient contents supplied from the ingredient, therefore, do not equate to the amounts available to the cells for metabolism of the animal and their utilization can only be evaluated in animal studies.

### 2.3 Table values

Feed formulation matrices in much of the poultry industry are reliant on and use tabulated values. These table values are global averages of published values for nutrient contents from local as well as world-wide sources. Number of such databases are available from different organizations and countries; examples include WPSA (1986), NRC (1994), INRA (2002), CVB (2016), Evonik (2016), FEDNA (2017), Feedipedia (2017) and Rostagno et al. (2017). For anyone perusing and comparing these databases, the inconsistency that exists among them will soon become clear. For example, AME values of 9.92, 9.71, 9.04, 9.80, 9.55 and 9.71 MJ/kg have been reported for soybean meal in NRC (1994), INRA (2002), CVB (2016), Evonik (2016), Rostagno et al. (2017) and FEDNA (2017), respectively. This variation may be explained inter alia by differences in chemical composition, presence of the anti-nutritional factors (ANF), age and breed of birds and the experimental methodology (Mateos et al., 2018).

In the development of table values, the local data is often combined with those from elsewhere, though it is recognized that the climate, cultivars and growing conditions that are unique to local conditions can influence nutrient contents. The variability can be high for non-conventional feed ingredients (Mateos et al., 2018). Most feed companies currently make use of table values, but often modifying them based on local analytical data, practical experience or NIRS data, to develop their exclusive nutrient and energy matrices.

### 2.4 Prediction equations

An array of prediction equations can be found in the literature and almost all are targeted towards the estimation of the metabolizable energy (ME) or AME, for poultry, of compound feeds from their chemical composition (Carpenter and Clegg, 1956; Sibbald, 1980; Carré et al., 1984, Carré et al., 2013). These equations are of limited value and confusing when extrapolated to predict the available energy of single feed ingredients. While some general equations are available for complete feeds, there are less equations available for raw materials. Janssen (1989) published a table with equations for the main raw materials available and this remains a major source of information. These equations have not been updated and, given the progress in plant genetics and poultry industry over time, their validity is limited. During the recent past, prediction equations have been proposed for the AME and true metabolizable energy (TME) of specific feed ingredients based on proximate analysis (Batal and Dale, 2006; Alvarenga et al., 2011; Meloche et al., 2013, 2014). Although these equations successfully predict the energy content for the ingredient samples used in the model development, their accuracy has not been validated for other batches of samples.

In general, the use of historical prediction equations for routine evaluation is not practical for several reasons. Firstly, most equations are based only on the nutrient composition of feed ingredients. This limits their usefulness for ingredients with thermo-labile ANF such as soybean meal and rapeseed meal, which are over- or under-processed (Mateos et al., 2018). Secondly, the assumption that proteins, starch and lipids are equally digestible, and their digestibility is constant, is incorrect (Cerrate et al., 2019). Thirdly, no matter how precisely they are derived, they do not have universal application particularly when predicting AME of individual ingredients as opposed to formulated diets (Farrell, 1999). Fourthly, nutritional value of feed ingredients, birds’ requirements and management change over the years. Moreover, analytical errors can affect the reliability of equations.

Ideally, the variable constituents of an equation should come from simple analytical procedures. Examples of prediction equations to determine energy content of feed ingredients and complete diets for poultry are presented in Table 1. Carré (1991) found that the inclusion of cell-wall related parameters could improve the accuracy of prediction equations for AME based on proximate components. Generally, crude protein, ash, fat, starch, sugars and sometime a fiber criterion such as crude fiber are important analytical parameters. For the prediction of the digestibility of nutrients, much less equations are available, primarily because there is seldom a robust relationship between digestibility and proximal composition. Recently, Cerrate et al. (2019) suggested series of equations to predict the energy value from digestible nutrients and established equations for AME and net energy (NE) of mixed diets.

**Table 1:** Estimating the energy value of feed and feed ingredients from chemical composition and digestibility values.

Ingredient	Prediction equation	Reference
Corn	MEn = 36.21 (CP) + 85.44 (EE) + 37.26 (NFE)	Janssen (1989)^1^
Sorghum	MEn = 31.02 (CP) + 77.03 (EE) + 37.67 (NFE)	
Wheat	MEn = 34.92 (CP) + 63.10 (EE) + 36.42 (NFE)	
Soybean meal	MEn = 36.63 (CP) + 77.96 (EE) + 19.87 (NFE)	
Distillers dried grains with solubles	TMEn = 2,732.7 + 36.4 (fat) - 76.3 (fiber) + 14.5 (protein) - 26.2 (ash)	Batal and Dale (2006)^2^
Distillers dried grains with solubles	AMEn = 3,517 + 46.02 (EE) - 82.7 (ash) - 33.27 × (HC)	Rochell et al. (2011)^3^
	AMEn = −30.19 (NDF) + 0.81 (GE) - 12.26 (CP)	
Distillers dried grains with solubles	AMEn = −12,282 + 2.60 (GE) + 89.75 (CP) + 125.80 (starch) - 40.67 (TDF)	Meloche et al. (2013)^3^
	AMEn = −14,322 + 2.69 (GE) + 117.8 (CP) + 149.41 (starch) - 18.30 (NDF)	
Corn	AMEn = 4,021.8 - 227.55 (ash)	Alvarenga et al. (2013a)^4^
	AMEn = 36.21 (CP) + 85.44 (EE) + 37.26 (NFE)	
Soybean meal	AMEn = −822.33 + 69.54 (CP) - 45.26 (ADF) + 90.81 (EE)	
	AMEn = 37.5 (CP) + 46.39 (EE) + 14.9 (NFE)	
Corn and soybean meal	AMEn = 4,164.187 + 51.006 (EE) - 197.663 (ash) - 35.689 (CF) - 20.593 (NDF)	
General	AMEn = 4,164.187 + 51.006 (EE) - 197.663 (ash) - 35.689 (CF) - 20.593 (NDF)	Alvarenga et al. (2015)^4^
General	ME = [18.03 (CP_digestible_) + 38.83 (fat_digestible_) + 17.32 × (NFE_digestible_)]/1,000	CVB (2016)^5^
Barley	ME = [9,258 - 9.258 (ash) + 7.709 (starch)]/1,000	
Oats	ME = [12,980 -12.98 (ash) + 48.82 (fat) - 25.50 (CF)]/1,000	
Wheat products (excluding wheat)	ME = [16,780 - 16.78 (ash) - 69.20 (CF)]/1,000	
Meat meal and meat and bone meal	ME = [14,200 - 19.15 (ash) + 25.1 (fat)]/1,000	
Soybean meal	ME = [7,690 - 7.69 (ash) + 6.464 (CP) + 29.43 (fat) - 16.09 (CF)]/1,000	
Vegetable feed ingredients	ME = 4.31 (CP_digestible_) + 9.29 (EE_digestible_) + 4.14 (NFE_digestible_)	Rostagno et al. (2017)^6^
Animal feed ingredients and fats	ME = 4.31 (CP_digestible_) + 9.29 (EE_digestible_)	

CF = crude fiber; CP = crude protein; EE = ether extract; GE = gross energy; HC = hemicellulose; ME = metabolizable energy; MEn = metabolizable energy corrected for nitrogen; NFE = nitrogen-free extract; NDF = neutral detergent fiber; TDF = total dietary fiber.

1 MEn unit, kcal/kg DM; component unit, % (DM basis).

2 TMEn unit, kcal/kg as-fed; component unit, % (as-fed basis).

3 AMEn and GE unit, kcal/kg DM; component unit, % (DM basis).

4 AMEn unit, kcal/kg DM; component unit, % (DM basis).

5 ME unit, MJ/kg DM; digestible and total content unit, g/kg (DM basis).

6 ME unit, kcal/kg DM; digestible CP, EE and NFE unit, g/kg (DM basis).

Clearly, more research is warranted to develop robust equations and they should be based on simple analytical variables and digestibility values.

### 2.5 Near-infrared reflectance spectroscopy

Near-infrared reflectance spectroscopy is a valuable technique that is already in widespread use in feed mills to measure proximate constituents (moisture, protein and fat) and detergent fiber. Calibration service is also provided in specialized laboratories, aligned to commercial feed additive companies, for the prediction of gross contents of amino acids (AA) and phytate. This technique is based on the absorption of infrared radiations by the chemical bonds in organic matter. Apart from its rapidity, NIRS is a physical non-destructive method, requiring minimal sample preparation and chemical reagents, with high accuracy. All these factors leading this method to be one of the most widely used (van Kempen and Bodin, 1998; Pujol et al., 2007; Owens et al., 2009).

Although NIRS relies basically on chemical bonds, the spectra can sometimes be related to more complex parameters due to the relationships between these parameters and specific chemical (or chemico-physical) properties of the sample. This is the background for the potential use of NIRS to predict energy content or digestibility parameters. For prediction of ME with NIRS, a direct calibration is needed with reference in vivo energy values from many feed samples. Early study by Valdes and Leeson (1992a), calibrated ME on 80 poultry feeds. The main factor of variation of ME in their study was fat content, which was well predicted by NIRS. In another study, Valdes and Leeson (1992b) calibrated ME on 49 feed ingredients, but some of the samples were out of the prediction, which proved that the database was not large enough. Other studies also have shown that NIRS method can predict the energy values of starch- and fiber-concentrated ingredients for roosters (Losada et al., 2009) and broiler chickens (Owens et al., 2009). However, the AME values of several raw materials were not well predicted from NIRS, neither when using specific equations or when extrapolating equations derived from complete diets (Valdes and Leeson, 1992d, 1994; Garnsworthy et al., 2000). Garnsworthy et al. (2000) reported that the AME of wheat samples was poorly predictable (*r =* 0.52) by NIRS. However, Losada et al. (2010) compared 3 regression equation models for predicting the AME of oilseeds and oil seed by-products for broilers, based on chemical composition, in vitro digestibility and NIRS. They found that the repeatability of chemical and in vitro data was inferior than the NIRS. Prediction from NIRS spectra had lower standard error of calibration and lower standard error of cross-validation values than those obtained by the other 2 methods.

While NIRS is economical, rapid and widely used to predict chemical composition of feed and feed ingredients, using NIRS for the prediction of digestibility is more complicated as it requires a large number of reference data. Garnsworthy et al. (2000) reported that ileal nitrogen digestibility was poorly predictable (*r =* 0.22) by NIRS. Owens et al. (2009) also found that the predictability of protein digestibility was highly variable (*r =* 0.23 to 0.76) in wheat using the NIRS method. To the authors' knowledge, few equations are available for digestibility of nutrients in complete feeds. Recently, Coulibaly et al. (2013) published a study with data on starch and protein digestibility, with a moderate precision of NIRS prediction for these 2 parameters when they were predicted from feed spectra. The residual standard errors of prediction were 2.75% and 3.41% for starch and protein digestibility, respectively. Nevertheless, for individual ingredients, it might be different, particularly in the case of products which thermal treatment can have determining effect on digestibility such as soybean meal. However, Cozannet et al. (2010) found that NIRS can predict protein or AA digestibility of cereal distiller's grains with strong thermal treatments, as the damage to protein and AA is linked to major changes in properties (color, physic–chemical properties of proteins, Maillard products).

There are several limitations associated with the use of NIRS technology. Firstly, setting up the NIRS system is costly and secondly, the technique needs accurate, careful and ongoing calibration (Patience et al., 2009). Thirdly, the quality of NIRS predictions is dependent on the accuracy and repeatability of reference values used for the calibration (Leeson 1997; Owens et al., 2009). These reference data must come from in vivo digestibility assays, which makes it dependent on animal studies on a continuous basis (Jha and Tiwari, 2016). Fourthly, this technique requires statistical expertise to calibrate and validate the results (Jha and Tiwari, 2016). Furthermore, NIRS penetrates deep into the sample due to its wavelength and the depth of penetration depends on the particle size and particle density (De Thomas and Brimmer, 2002). Thus, not only the feed ingredient or complete feed type but also their other physical properties need to be considered (Jha and Tiwari, 2016). Although, in NIRS technique several quality parameters can be analyzed simultaneously but, it is unable to determine distribution of chemical constituents within the sample (Wu et al., 2008; Kumar et al., 2016). There is new technique called hyperspectral imaging (HSI) or imaging spectroscopy which combines properties of imaging and spectroscopy. The HSI technique is able to provide simultaneous detection and quantification and localization of chemical constituents of the sample. Due to combined features of imaging and spectroscopy, HSI has become a promising technique for non-destructive food quality, as well as, safety analysis, particularly in the meat industry, the technology has gained significant attraction (Kumar et al., 2016).

In general, NRIS method compared to other analytical methods is more rapid and is able to estimate multiple constituents of each sample in a single measurement in a real-time basis. An added advantage of the method is that the calibration developed in one NIRS instrument can be transferred to other NIRS instruments in the field through a process called calibration transfer (Fernandez-Ahumada et al., 2008; van Kempen and Simmins, 1997). Ideally, wider the range of input data better will be the calibration (van Kempen and Simmins, 1997). It should be noted, however, that the NIRS method still require data from chemical analysis, in vivo and in vitro studies, so this method may not be considered independently in feed evaluation.

### 2.6 In vivo assays

In vivo methods measure the direct animal response to variation in diet and is the best method to determine the nutritional value of feed ingredients.

#### 2.6.1 Energy evaluation

An ideal energy system must be easy to measure, predictive of bird performance, additive in feed formulations and independent of bird factors. However, energy metabolism is too complex to meet all these ideals. Since the 1950s, the AME has been the system of choice of describing available energy for poultry (Hill and Anderson, 1958). True metabolizable energy became popular in the 1980s but has since lost favor owing to welfare concerns. Currently, the AME is the widely accepted system to describe available energy and will remain the favoured system in the foreseeable future. It is not a perfect system, with number of limitations (Mateos et al., 2018; Wu et al., 2020). But it is easy to measure, familiar and universal, and these features have put the AME well ahead of other energy measurements.

A major concern with the large body of available AME data is the wide variability reported. There are 2 sides to the observed variation, namely (1) inherent variability expected in raw materials and (2) the differences arising from methodological differences employed in different research stations. The ramification is that the AME measurement need be improved by standardizing the methodology and then the actual ingredient variation can be measured and AME across laboratories will become comparable. For example, Black et al. (2005) used a standardized methodology, in terms of assay diets, age and strain of birds, grain processing and laboratory analysis, to assay large number of samples from 5 cereal grains to delineate the actual sample differences. Recently, Wu et al. (2020) proposed the standardization of the procedures that are used in the in vivo trials.

Net energy system, a refinement of the AME concept, has received attention from time to time (Swick et al., 2013; Wu et al., 2020). In theory, NE will more closely describe the energy available in an ingredient for bird's metabolic functions and is more predictive of animal performance. It is, however, difficult assay, costly and time consuming. Its economic advantage over the AME system is well demonstrated in pigs and ruminants, but not in poultry (Zuidhof, 2019).

#### 2.6.2 Protein evaluation

A century ago, poultry feeds were mixed on the basis of crude protein. With advances in analytical chemistry, the stage was set during the 20th century for a shift towards the use of total AA (Elwinger et al., 2016). During the past 30 years, the basis of feed formulation has gradually shifted to the digestible AA system, which has enabled us to meet AA requirements more precisely and to increase the range and inclusion levels of alternative ingredients, while maintaining performance levels. Initially, the AA digestibility measurement was based on excreta analysis. During the 1980s, the precision-fed rooster assay (Likuski and Dorrell, 1978) was popular because it was easier to conduct and rapid. Over the years, however, this assay has slowly lost global acceptance owing to ethical issues associated with prolonged fasting. Another concern with this assay was physiological changes and changes in the secretion of digestive enzymes as fasting of birds do not represent “normal” feeding behaviour (Lemme et al., 2004).

During the past 2 decades, ileal-based broiler digestible AA is being increasingly accepted and has become the norm (Ravindran and Bryden, 1999; Lemme et al., 2004); although shifting from excreta-based values to ileal-based values initially needed considerable convincing. Considerable published data have now become available for the ileal AA digestibility of raw materials. A major issue with these data, however, is the wide variability reported for digestibility estimates, which is due partly to differences in methodology. This is a real concern, precluding true comparison of data from different laboratories and exemplified by the results from a recent collaborative study (Ravindran et al., 2017). In phase 1, pronounced variations in the ileal AA digestibility of corn were observed when the assay was conducted using station protocols, differing in methodology (Table 2). In phase 2, use of a common agreed protocol, in which a corn-soybean meal diet was assayed, eliminated the variation among 5 research stations in apparent AA digestibility (Table 3). Overall, these results highlight the need for a consensus protocol to use in the measurement of ileal AA digestibility of raw materials for poultry, which will minimize methodological effects and enable better comparison of data generated across research stations. Based on these data and published works, a standard protocol was proposed for the determination of AA digestibility in feed ingredients for broiler chickens (Ravindran et al., 2017).

**Table 2:** Apparent ileal digestibility coefficients of crude protein (CP) and essential amino acids (AA) in corn determined at 3 research stations, using station protocols^1^.

Item	Station 1	Station 2	Station 3	SEM	*P*-value
CP	0.73	0.85	0.80	0.027	0.05
Arginine	0.80	0.91	0.91	0.022	0.01
Histidine	0.79	0.89	0.85	0.026	0.06
Isoleucine	0.75	0.88	0.79	0.040	0.11
Leucine	0.87	0.93	0.87	0.023	0.13
Lysine	0.63	0.83	0.79	0.036	0.05
Methionine	0.84	0.92	0.86	0.029	0.16
Phenylalanine	0.79	0.90	0.83	0.031	0.08
Threonine	0.62	0.77	0.73	0.023	0.05
Tryptophan	0.61	0.80	0.80	0.049	0.06
Valine	0.75	0.87	0.81	0.034	0.07
Average^2^	0.76	0.87	0.83	0.030	0.05

1 From Ravindran et al. (2017).

2 Average of 17 AA.

**Table 3:** Reduced variation in apparent ileal digestibility coefficients of crude protein (CP) and essential amino acids (AA) of corn-soybean meal diet for broilers determined in 5 research stations, using an agreed protocol^1^.

Item	Range	CV, %	*P*-value
CP	0.84 to 0.86	1.1	0.42
Arginine	0.90 to 0.92	1.1	0.60
Histidine	0.87 to 0.89	1.0	0.27
Isoleucine	0.85 to 0.87	1.2	0.51
Leucine	0.87 to 0.89	0.9	0.37
Lysine	0.86 to 0.89	1.4	0.53
Methionine	0.89 to 0.91	1.2	0.37
Phenylalanine	0.81 to 0.87	2.7	0.16
Threonine	0.78 to 0.82	2.1	0.20
Valine	0.84 to 0.86	1.4	0.30
Average^2^	0.84 to 0.87	1.1	0.33

1 Ravindran et al. (2017).

2 Average of 17 AA.

A large volume of published values, including several compilations (e.g. Sibbald, 1986; Parsons, 1991; Bryden et al., 2009; Evonik, 2016; Blok and Dekker, 2017) on AA digestibility coefficients for poultry are now available. However, confusion about the terminology used to describe the AA digestibility estimates becomes clear to anyone perusing the available digestibility data. For each AA, there are at least several possible values, and combinations thereof, to describe the digestibility for poultry: apparent, true or standardized; adult rooster or broiler; excreta or ileal. Currently, true/standardized ileal digestibility is becoming the norm in poultry feed formulations.

Despite providing the true measure of nutritional value, in vivo trials have their obvious limitations; they are costly in terms of equipment and personnel, require plenty of feed materials and animals, the number of feed ingredients that can be evaluated is limited, they are time-demanding, for some measurements surgical interventions are necessary, and finally there are growing welfare-related unease regarding the use of animals. These are the impetus behind the interest in testing and development of in vitro evaluation techniques.

### 2.7 In vitro digestion methods

The concept of in vitro digestion assays was initially developed for ruminant feeds as an alternative to the costly, labour-intensive and time-consuming in vivo methods to predict the nutrient digestibility, and subsequently actively pursued in pig research. An edited book by Fuller (1991) provides an excellent coverage of early research on in vitro assay methods in pigs and poultry. There have also been several authoritative reviews since (Farrell, 1999; Moughan, 1999; Butts et al., 2012), which provide useful background information to the current review.

Tilley and Terry (1963) were the pioneers of the original work proposing a 2-stage rumen fluid-pepsin technique for the prediction of organic matter digestibility in forages for ruminants. Since then, in vitro methods have evolved and been successfully applied to pigs (Boisen and Eggum, 1991; Boisen and Fernández, 1997; Noblet and Jaguelin-Peyraud, 2007) and to a less extent in poultry feeds. In poultry, in vitro methods have been used to evaluate either energy (Clunies et al., 1984; Valdes and Leeson, 1992c; Losada et al., 2009, 2010; Palić et al., 2012) or protein (Sakamoto et al., 1980; Clunies and Lesson, 1984; Fru-Nji et al., 2011). Several advantages of using in vitro digestion methods over in vivo methods can be mentioned. Firstly, the in vitro digestion method is rapid and less expensive. Secondly, the animal variability effects are removed. Thirdly, in vitro digestion methods enable the simulation of gastrointestinal tract (GIT) segments and finally, in vitro digestion methods overcome the ethical concerns related to animal research. Other influencing factors such as management, environment, disease and genotype are also removed in in vitro evaluations.

In vitro methods should be designed to simulate digestive processes in the GIT of the animals, as closely as possible (Boisen and Eggum, 1991; Longland, 1991; Huang et al., 2000).

As indicated earlier, almost all current in vitro models for monogastrics are based on pigs (Furuya et al., 1979; Boisen and Fernández, 1995, Boisen and Fernández, 1997) and commonly used to generate in vitro data for poultry (Sakamoto et al., 1980; Clunies and Leeson, 1984; Clunies et al., 1984; Losada et al., 2009, 2010). Pig and poultry are both monogastric species and, their digestive physiology, absorption and transport of nutrients in the small intestine are essentially similar to a large extent. There are, however, obvious anatomical differences in the GIT, with the presence of crop and gizzard in poultry and longer, well-developed hindgut in pigs. These dissimilarities lead to differences in the transit time and pH of digesta along the digestive tract and, modify the foregut digestion in poultry and hindgut fermentation in pigs (Table 4, Table 5). To the authors’ knowledge, there are no reports comparing the digestibility in pigs and poultry, but there are indirect evidence suggesting that they are dissimilar (Donkoh et al., 1994; Ravindran, 2013). For these reasons, there is a need to consider the differences in digesta retention time and pH in the development of appropriate in vitro assays in poultry.

**Table 4:** The pH, transit time (min) and relative length (cm/kg body weight) of different segments of the digestive tract of broiler chickens.

Segment	pH^1^	Transit time^1^	Relative length^2^
Crop	5.5	10 to 50	–
Proventriculus + gizzard	2.5 to 3.5	30 to 90	–
Duodenum	5.0 to 6.0	5 to 10	22.5
Jejunum	6.5 to 7.0	20 to 30	56.2
Ileum	7.0 to 7.5	50 to 70	60.1
Small intestine	5.0 to 7.5	75 to 110	139
Cecum	8.0^3^	20 to 30^3^	13.7

1 From Ravindran (2013).

2 From Abdollahi et al. (2013a).

3 Cecum + colon.

**Table 5:** The pH, relative length (%), relative capacity (%) and transit time (h) in different segments of the digestive tract of pigs.

Segment	pH^1^	Relative length^1^	Relative capacity^1^	Transit time (solid phase)^2^	Transit time (liquid phase)^2^
Stomach	2.2	–	29.2	1.1	0.8
Small intestine	6.0 to 7.5	78	33.5	3.9	4.0
Cecum	6.3	1	5.6	–	–
Colon	6.8	21	31.7	39.0	36.0

1 From Kararli (1995).

2 From Wilfart et al. (2007).

It is clear that the in vivo conditions cannot be completely simulated under in vitro conditions, due to the complexity of in vivo digestion. An ideal in vitro method should be simple, rapid, accurate and reproducible to predict in vivo responses. The in vitro data should be validated by comparing with corresponding data collected from in vivo studies using the same samples. In a successful in vitro technique, there will be a strong correlation between the estimates (Sakamoto et al., 1980; Clunies and Leeson, 1984; Valdes and Leeson, 1992c; Graham, 1991; Boisen and Fernández, 1995) and appropriate statistical analyses should be used to determine the accuracy and precision of prediction equations of in vitro data relative to in vivo results. These statistical analyses can provide information such as coefficient of determination (*R*^2^), residual standard deviation (RSD), and standard error of prediction (SEP) values. In general, prediction equations with high *R*^2^ and low RSD or SEP values are reliable (Furuya et al., 1979; Clunies et al., 1984; Regmi et al., 2008, 2009), as these values are considered as precision of prediction of the in vivo values from the in vitro results.

A myriad of in vitro digestion methods has been reported over the years. All methods are based on the measurements of the insoluble and undigested material collected after filtration or centrifugation, which could be categorized into 1-, 2- or 3-step digestion systems. One-step digestion model is commonly used to simulate nutrient digestion in the gastric phase (Ehle et al., 1982; Holzgraefe et al., 1985) and the 2-step model is used to simulate the gastric and small intestinal phases (Furuya et al., 1979; Clunies et al., 1984; Clunies and Leeson, 1984). The 3-step model mimics the digestion and fermentation in the gastric phase, small intestine and large intestine, accounting for nutrient disappearance over the entire digestive tract (Boisen and Fernández, 1997; Regmi et al., 2008, 2009). This model is more applicable to pigs and not to poultry, wherein the hindgut is shorter, and the microbial fermentation is less significant. In the 3-step in vitro digestion method, the indigestible carbohydrates are degraded using either purified fiber-degrading enzymes or inoculum containing live microbes (Williams et al., 2005). The former is used in assays investigating the digestibility of organic matter or energy, and the latter is used in assays measuring the fermentability in terms of gas or short-chain fatty acid production, or disappearance of organic matter or non-starch polysaccharides (NSP) (Wang, 2014). These models yield different end-products, with simple sugars or low molecular weight polymers for enzymes and short-chain fatty acids and gases for inoculum (Jørgensen et al., 1997).

The 2 commonly used purified enzymes are Viscozyme (Novo-Nordisk, Bagsvaerd, Denmark; Boisen and Fernández, 1997), containing a cocktail of enzymes (arabinase, cellulase, B-glucanase, hemicellulase, xylanase and pectinase), which are capable of degrading fiber components and cellulase (Huang et al., 2003). Cellulase, is obtained from specific bacterial species such as *Aspergillus spp.* or *Trichoderma viridae* (Regmi et al., 2008).

In monogastric animals, ingested fibers that are not digested by endogenous enzymes become available for microbial fermentation, mainly in the large intestine and produce metabolites like volatile fatty acids (acetate, propionate, butyrate). However, poultry are an exception, as retention time in the caeca is only around 20 to 30 min (Ravindran, 2013). The microbial inoculum to initiate in vitro fermentation in pigs can be obtained either from the cecum, rectum, or feces, and cecal content can be applied as inoculum for poultry in vitro fermentation studies (Jha and Tiwari, 2016). Pig fecal inoculum has also been used to simulate digestion of fiber in broiler diets (Marrero et al., 1998). It is assumed that gas and metabolites produced during in vitro fermentation reflects the same kinetics and metabolite production as in vivo fermentation of fiber in the large intestine of animals. The validation of the results from in vitro fermentation techniques with in vivo studies is important. Three-step in vitro digestion methods have been successfully employed to predict energy digestibility of feed ingredients and complete diets for pigs (Beames et al., 1996; Boisen and Fernández, 1997; Huang et al., 2003; Regmi et al., 2008, 2009) and the use of the purified enzyme, viscozyme, to estimate digestible energy (DE) and organic matter seemed promising (Boisen and Fernández, 1997; Regmi et al., 2008, 2009).

## 3 Application of in vitro methods for poultry

The 2-step digestion model employing multiple enzymes is an improvement over chemical analyses to predict the nutritional value with greater accuracy in pigs (Boisen and Fernández, 1997; Noblet and Jaguelin-Peyraud, 2007; Valdes and Leeson, 1992c). In poultry, however, data based on in vitro techniques are limited and contradictory (Clunies and Leeson, 1984; Clunies et al., 1984; Valdes and Leeson, 1992b; Losada et al., 2009, 2010). Valdes and Leeson (1992c) used the 2-step digestion method (pepsin treatment followed by pancreatin, bile salts and enterokinase treatment) to predict the AME of poultry diets and reported a high accuracy (*r =* 0.84) for prediction. However, they concluded that in vitro digestion method applied in their study had limited applications and cannot be used universally to estimate AME in compound feeds. Losada et al. (2009) found that the AME prediction accuracy, based on in vitro digestibility of DM and organic matter, were lower compared to predictive equations based on NIRS method. In contrast, Clunies and Leeson (1984) found that in vitro DM and protein digestibility were highly correlated (*r =* 0.99 and 0.93, respectively) with in vivo ileal digestibility obtained with 7-wk-old broilers. It was also noted that in vitro data obtained with highly digestible diets predicted in vivo DM digestibility better than those with poor digestibility and suggested that fermentation may have been significant in diets with poorer digestibility.

Commercial poultry feeds are a mixture of several ingredients with different inclusion rates, which make the use of in vitro technique more complicated. Each ingredient has different characteristics and, for this reason, the optimum pH, enzyme concentrations and incubation duration may not be attained in the in vitro system for all feed components (Clunies and Leeson 1984; Valdes and Leeson 1992c). Valdes and Leeson (1992c) suggested that, taking these differences into account, a specific in vitro digestibility assay should be developed for each feed ingredient. Several reports are available on correlations between in vitro and in vivo data in pigs (Furuya et al., 1979; Boisen and Fernández, 1995, Boisen and Fernández, 1997; Williams et al., 2005; Regmi et al., 2008, 2009), but this is not the case in poultry. Moreover, comparisons in poultry have focussed on total tract digestibility determined with adult roosters (Clunies et al., 1984; Losada et al., 2009, 2010; Palić et al., 2012), which are not applicable to growing broilers. For instance, Svihus and Gullord (2002) found that the AME of diets for adult roosters were higher compared with that for broilers.

In vitro digestion methods are being continuously refined, but yet to be completed or validated against in vivo data in poultry. To accomplish this, some main requirements need to be researched and satisfied, including; matching of in vitro enzymes with in vivo enzymes, enzyme-to-substrate ratios, pH, temperature, incubation time for each digestion phase, sample size, size and distribution of particle. Every effort should be made to simulate digestive processes in the GIT of poultry, as closely as possible.

Two published in vitro models to predict the AME (Valdes and Leeson, 1992c) and protein digestibility (Clunies and Leeson, 1984) of poultry diets are described below in detail.

### 3.1 In vitro prediction of AME

This 2-stage digestion system for AME prediction, developed by Valdes and Leeson (1992c) involves measurement of the gross energy of the diet sample and the undigested residue. In brief, half a gram of ground sample is incubated with pepsin solution (containing 20 mg pepsin, 11,400 units) at 37 °C and pH 4.13 for 4 h in an agitating water bath. After completion of this stage of digestion, the pH is adjusted between 7.0 and 7.1 with sodium hydroxide solution. An enzyme solution containing pancreatin, bile salts and enterokinase is added. The next stage of digestion then proceeds for 6 h at 37 °C. At the end of the second stage of incubation, samples are centrifuged (1,500×*g* for 15 min) and the supernatant is discarded. The undigested precipitate is washed with double-distilled water, centrifuged again, and supernatant discarded. The residue is assumed to be the indigestible matter. These residues are dried in an oven at 65 °C for 48 h and weighed. The residue and the original feed sample analyzed for gross energy and the in vitro digestible energy is calculated according to the following formula:

In vitro digestible energy (kcal/g) = [(GE _Feed_ × *F*) – (GE _Residue_ × *R*)]/*F*,

where GE _Feed_ = gross energy of feed (kcal/g), *F* = weight of feed (g), GE _Residue_ = gross energy of residue (kcal/g), and *R* = weight of residue (g).

### 3.2 In vitro prediction of protein digestibility

A procedure, modified from the one proposed by Furuya et al. (1979), was used by Clunies and Leeson (1984) for the prediction of DM and protein digestibility in poultry. In brief, half a gram of ground sample is incubated with pepsin solution (containing 20 mg pepsin, 11,400 units) at 37 °C and pH 4.13 for 4 h in an agitating water bath. After completion of the first stage of digestion, the pH is adjusted between 7.0 and 7.1 with sodium hydroxide solution and porcine intestinal fluid is added. The second stage of digestion then proceeds for 4 h at 37 °C. At the end of the second stage of incubation, samples are centrifuged (1,250×*g* for 10 min at a temperature of 5 °C) and supernatant discarded. The undigested precipitate is washed with distilled water, re-centrifuged and the supernatant again discarded. The residue is assumed to be the indigestible matter. The low temperature at which the final incubate is centrifuged is the means by which the action of the added enzymes is stopped. The undigested precipitate is then transferred to a dry pre-weighed filter paper for DM and protein determinations. In vitro DM and protein digestibility are determined using the following formulas:

In vitro DM digestibility (%) = [(DM _Feed_ – DM _Undigested_)/ DM_Feed_] × 100,

In vitro protein digestibility (%) = [(Protein _Feed_ – Protein _Undigested_)/ Protein _Feed_] × 100,

where DM _Feed_ = gram DM in 0.50 g of sample, DM _Undigested_ = gram DM precipitate, Protein _Feed_ = gram protein in 0.50 g of sample, and Protein _Undigested_ = gram protein in precipitate.

## 4 Other in vitro methods

Several other in vitro assay methods are also available: dialysis cell method, pH-drop and pH-stat methods, immobilized digestive enzyme assay (IDEA) and computer-controlled method. These methods are employed for the evaluation of protein quality, mainly of human foods. The solubility index method and dispersibility index are 2 methods used to evaluate the quality of protein in poultry diets. For details of these methods, refer to reviews by Boisen and Eggum (1991), Butts et al. (2012) and, Bryan and Classen (2020).

Gauthier et al. (1982) developed an in vitro method under constant dialysis (molecular weight cut off 1,000 Da) with specialized apparatus (dialysis cell method) to address the concern that enzyme activity is reduced by the products of digestion. Boisen and Eggum (1991) suggested that the dialysis cell method is valuable to study luminal protein degradation in the small intestine to predict the AA availability. However, the method is time consuming, taking 5 d to complete and needs complex equipment.

In the pH-drop method, it is assumed that the pH drop is correlated with protein hydrolysis and protein digestion (Mozersky and Panettieri, 1983). The pH-drop method has been found to be highly correlated with apparent total tract protein digestibility (Hsu et al., 1977; Satterlee et al., 1981) and true ileal protein digestibility (Moughan et al., 1989) in rats. Porter et al. (1984) suggested that the pH-drop method of Hsu et al. (1977), which has a very short period of incubation with enzymes, may result in the underestimation of the digestibility of structurally stable proteins. A modified method of pH-drop is the pH-stat approach, which was developed by Pedersen and Eggum (1983). In this method, pH is kept constant by automatic titration with 0.1 mol/L sodium hydroxide. The amount of alkali used to maintain the pH constant is recorded at the end of the incubation. Compared with the pH-drop method, the pH stat method was reproducible and highly correlated with in vivo total tract digestibility in rats for plant (*r =* 0.85) and animal protein sources (*r =* 0.92). Boisen and Eggum (1991) stated that this approach provides a better prediction of protein digestibility and that the same regression equation could be applied for variety of feed materials. In caecectomized cockerels, there was a good correlation with lysine digestibility in caecectomized cockerels and the pH drop test.

Immobilized enzyme assay was developed by Porter et al. (1984) and Swaisgood and Catignani (1985). This system appeared to be an accurate and reliable estimate of in vivo total tract protein digestibility in rats (*r =* 0.83) for a range of feed ingredients and diets (Chang et al., 1990). A digestive enzyme assay kit (Poultry Complete IDEA, Novus International Inc., St. Charles, MO) is now commercially available for the prediction of true total tract AA digestibility of animal protein meals and soybean meal (Schasteen et al., 2002, 2007). But this calibration is based on in vivo values determined with precision-fed adult roosters. Originally Minekus et al. (1995) and later Wickham et al. (2009) designed a multi-compartmental, computer-controlled model which simulates the dynamic events occurring within the GIT of human and monogastric animals. The model closely reproduces the physiological functions of GIT, such as peristaltic movements, pH, gastric and intestinal secretions, GIT transit, and absorption of digested products and water. It, however, suffers from 2 key drawbacks. First, it is costly to set up and maintain and has a low throughput and not useful for routine evaluation of feed ingredients. Second, it still does not mimic the physiological processes of gut wall such as active transport and neural and hormonal feedback mechanisms (Minekus et al., 1995; Yoo and Chen, 2006).

In protein solubility tests, either sodium or potassium hydroxide or sodium tetraborate can be used as the alkali solution (Parsons et al., 1991; Lee et al., 1992; Bryan and Classen, 2020). However, protein dispersibility test involves high speed mixing of a protein sample in water, followed by the assessment of solubility (Batal et al., 2000). Although solubility index and dispersibility index methods provide a rating of the protein quality of feed ingredients, but do not give any indication of the amount of nutrient absorbed by the animal. In general, protein solubility and protein dispersibility methods are used as measures of ingredient quality in most poultry nutritional research evaluating high protein ingredients (Bryan and Classen, 2020).

## 5 Factors affecting in vitro digestibility estimates

### 5.1 pH and incubation temperature

When in vivo ileal or total tract digestibility of nutrients is predicted by in vitro methods, the pH should not exceed the actual pH of the animal digestive tract (Eggum and Boisen, 1991). However, the pH in the GIT is variable depending on the diet composition, feed form, inclusion of whole grain, feeding level and the physiological status of the animal (Cone, 1993). The relevance of pH in enzyme activity and effectiveness of endogenous enzymes is well known (Ravindran, 2013).

The pH in the in vitro digestion system is critical for the degree of nutrient solubility and hydrolysis of feed ingredients (Cone, 1993). Pepsins have 2 optimum pH, one at pH 2.0 and one near 3.3 (Bottger and Holler, 1974). Löwgren et al. (1989) reported that the DM disappearance in the second stage of incubation at pH 6.9 was significantly higher compared to pH 5.9 with duodenal and faecal media. However, Clunies and Leeson (1984) reported pH (6.5, 6.6., 6.7, 6.8 and 6.9) had no effect on the protein digestibility in the second stage of incubation. They also did not observe any differences in the in vitro digestibility of DM at pH of 6.6 to 6.9. However, when the pH was reduced to 6.5, there was a significant decrease in DM digestibility. Based on these findings, a pH range of 6.6 to 6.9 was recommended for the second stage.

When selecting the appropriate pH, one must also consider that the products of digestion can decrease the pH (Clunies and Leeson, 1984). Because diets are likely to differ in their buffering capacity, the amount of acid or base required to obtain the desired pH may also differ from diet to diet. Cone (1993) determined the in vitro protein solubility of different feed ingredients at pH of 3.0, 6.0 and 9.0 and reported that the solubility was highest at pH 9.0 and lowest at pH 3.0 for most feed ingredients. However, they observed considerable variation in protein solubility between feed ingredients at different pH levels.

There is no published data examining the effect of incubation temperature on in vitro digestibility values. It is important to keep the incubation temperature as close as possible to the physiological temperature from 39 to 41 °C (Bennett et al., 1986; Annett et al., 2002).

### 5.2 Sample weight and particle size

Variable sample weights have been used in in vitro assays. For example, Steinhart and Kirchgessner (1973) used only 0.2 g of samples. On the other extreme, Fru-Nji et al. (2011) evaluated the effect of an exogenous protease on the in vitro protein digestibility using 10 g feed samples. Boisen and Fernández (1997) investigated the effect of 0.5 vs. 1.0 g of sample weights and observed that, in ingredients with high protein content (potato protein, soybean meal and peas), the in vitro digestibility of 1.0 g of samples was underestimated.

Particle size is an important factor that can affect the degree of enzyme access to substrate. During in vivo digestion, particle size is reduced through mastication (pigs) or gizzard action (poultry) but remains static in in vitro models. It is also known that the response to the particle size in in vivo digestion in poultry is variable depending on the type of ingredient (Amerah et al., 2008). Smaller particles in in vitro process have a larger surface area with greater enzyme access to substrates and subsequently resulting in higher digestibility (Clunies and Leeson 1984; Boisen and Fernández, 1997). Clunies and Leeson (1984) examined the effect of 3 particle sizes in mash and reground diets using screen sizes of 0.84 or 0.40 mm in in vitro digestibility and reported increased DM and protein digestibility for all diets with smaller particle size.

Löwgren et al. (1989) showed that the effect of screen size of 0.5, 1.0, 2.0 and 5.0 mm on DM disappearance in barley grain was insignificant when the incubation lasted longer than 45 h. They reported that large particles require a longer incubation time in vitro, but final digestibility values may not be affected. Several authors have studied the influence of particle size on the solubility and digestibility of protein in different feed ingredients. Dale (1990) evaluated the effect of 8 different particle sizes of dehulled soybean meal (184, 251, 299, 556, 599, 707, 831, 939 μm) on protein solubility. They found that as the mean particle size increased from 184 to 939 μm, protein solubility decreased from 90% to 70%. Parsons (1991) and Parsons et al. (1991) stated that the particle size should be kept consistent across in vitro assays, as varying particle sizes may influence the repeatability of results. Furuya (1991) also stated that in vitro protein digestibility in samples with a smaller particle size (0.5 mm) was higher than those with a larger particle size (1.0 mm). They also reported that the magnitude of changes varied with the type of ingredient. In corn samples with particle sizes of 1.0 and 0.5 mm, the in vitro protein digestibility was 73% and 83%, respectively, and the corresponding values for wheat were 90% and 91%, respectively.

In general, it can be concluded that in vitro digestibility increased with decreasing particle size. To achieve comparable digestibility across assays, the particle size of feed samples for in vitro models should be the same as or smaller than that used for in vivo studies (Wang, 2014).

### 5.3 Physical form of the sample

In a study by Noblet and Jaguelin-Peyraud (2007), the 3-step in vitro digestion model performed well for mash, but not for pelleted feeds. It was suggested that in vitro prediction equations created using mash diets may not provide an accurate estimate of the in vivo energy digestibility of pelleted diets in pigs. Pelleting is the most prevalent heat treatment in the production of poultry feed. Offering feed to poultry in pellet form enhances the economics of production by increasing the feed intake, and thus growth performance and feed efficiency. However, the process of making pelleted diets may also have detrimental effects on the production through chemical and physical changes that occur during pelleting (Abdollahi et al., 2013a,b). Pelleting-induced particle size reduction results in an under-developed gizzard, lowered secretion of hydrochloric acid that increases the pH of the digestive tract, and a reduction in digestive enzymes (Engberg et al., 2002; Abdollahi et al., 2013a). Moreover, feeding pelleted diets reduces the time that feed spends in the upper GIT, which can be a limiting factor for enzyme efficiency in pelleted diets (Zaefarian et al., 2016).

The influence of pelleting on the in vivo nutrient digestibility in poultry is being increasingly recognized (Abdollahi et al., 2011; Abdollahi et al., 2013a, Abdollahi et al., 2013b), whether similar effects occur under in vitro assay conditions is of future interest. These complex matrix of conditions (pH, retention time, endogenous enzyme secretion) need to be considered in in vitro assays.

### 5.4 Incubation time

Incubation times employed to simulate gastric and intestinal digestion vary greatly in in vitro assays and are not standardized. Because digestion is a function of enzyme activity and time, increased digestion time will increase the nutrient digestibility. Clunies and Leeson (1984) tested 1, 2, 3, and 4 h of incubation times for the first (pepsin) and second (intestinal fluid) stages of incubation in 2 experiments. For the first stage of incubation, DM and protein digestibility increased during the second hour of incubation, and no further increase was observed between 2 and 4 h. For the second stage of incubation, increases in DM digestibility were observed in each of the first 3 h of incubation but not in the last hour. Protein digestibility increased during the second h of incubation and remained constant afterwards. Losada et al. (2009) conducted a preliminary in vitro study to compare 8 incubation times increasing from 4 to 19 h with wheat and corn in the second incubation step and found that organic matter digestibility increased with time from 4 to 7 h, especially in the case of corn, and reached a plateau thereafter.

Overall, limited available data on the incubation time during in vitro gastric and intestinal phases is inconclusive. In poultry, transit time in the proventriculus/gizzard axis is 30 to 90 min and 75 to 110 min in the small intestine (Ravindran, 2013, Table 4). Svihus (2011) reported that the average retention time in the digestive tract, excluding the caeca, is around 3 to 4 h. It is evident, for best results, that incubation times used in pig in vitro models must be modified and aligned with GIT conditions in poultry.

### 5.5 Type and concentration of in vitro digestive enzymes

The type and concentration of enzymes used in the digestion process will obviously affect in vitro results, as the specificity of the enzymes defines which substrates and bonds are hydrolyzed. In the case of protein, the digestion is initiated by the action of pepsin during the first stage of incubation (Clunies and Leeson, 1984). Johnston and Coon (1979a) found that a 0.2% pepsin-HCl solution is excessive and will completely digest proteins of poor-quality ingredients such as meat and bone meal and feather meal. When the pepsin concentration was decreased by 10-fold to 0.02%, the digestible protein value for 9 meat and bone meals were almost identical to those obtained with 0.2% pepsin. However, when the pepsin concentration was reduced to 0.002%, the digestible protein was significantly reduced. In a subsequent study, these researchers found that with 0.002% pepsin concentration, digestible protein values were highly correlated with estimates of protein efficiency ratio and net protein utilization in meat and bone meals and feather meals (Johnston and Coon, 1979b). Similar results were reported for meat meal, feather meal and poultry by-product meal by Parsons (1991), who found digestible nitrogen values for feather meal and meat meal determined with 0.002% pepsin were highly correlated with in vivo rooster lysine digestibility values compared to those determined with 0.2% pepsin. For the poultry by-product meal, digestible nitrogen values from both pepsin concentrations were highly correlated with lysine digestibility values. It was suggested that the 0.002% pepsin concentration is superior to 0.2% pepsin in predicting in vivo protein and AA digestibility of meat meal and feather meal. Clunies and Leeson (1984), testing 3 activities of pepsin (290, 580 and 1,140 units per 10 mL of 0.075 mol/L HCl) in the first stage of incubation, found that increasing pepsin activity from 290 to 580 units increased the in vitro DM and protein digestibility. When the pepsin activity was increased to 1,140 units of enzyme, protein digestibility increased, and DM digestibility was unaffected. Further studies are warranted to investigate the effects of enzyme concentration and activity in poultry in vitro assays.

### 5.6 Ingredient type and presence of anti-nutritional factors

A major purpose of in vitro digestibility evaluation is to detect differences in nutrient digestibility and rank feed ingredients. For some feed ingredients, however, in vitro digestion models appear not to be feasible. In the studies of Graham and Löwgren (1991) and Noblet and Jaguelin-Peyraud (2007), data on rapeseed meal and lupin meal had to be excluded from statistical analysis as outliers because of the possible interference of ANF.

Feed carbohydrates can be partitioned into readily digestible (mainly starch) in the small intestine or indigestible (mainly NSP) that are fermented in the hindgut. These two components require different incubation times during the in vitro digestion process. The presence of NSP and ANF in feed samples can also affect in vitro enzyme activity (Schneeman, 1982). In by-product feeds, the presence of NSP adds a major impediment. A mechanism of inhibition by NSP is the absorption of enzymes into the cell wall matrix or unspecific bindings to the NSP (Schneeman, 1978). The reported reductions in enzyme activity in the multi-enzyme assay compared to single-enzyme assay lends credence to this thesis (Howard and Mahoney, 1989).

## 6 Factors that cannot be mimicked in in vitro digestion models

There are number of animal- and feed-related factors which cannot be accounted for in vitro assays. For instance, the effect of ANF (such as lectins, tannins and trypsin inhibitors) on digestion cannot be simulated in the in vitro method. All feed ingredients, with few exceptions, contain ANF which interfere with the digestion of nutrients (Hedemann et al., 1999; De Lange et al., 2000). However, as noted by Hsu et al. (1977), in vitro digestion models can be used to investigate the effects of purified forms of ANF and thermal treatment on the protein digestibility of ingredients. Eggum and Christen (1975) showed that the addition of 1% tannic acid to a soybean protein diet reduced the in vivo digestibility of protein by 6%, and 1% of tannic acid addition to a barley diet decreased the in vitro digestibility by 3% to 4% (Pedersen and Eggum, 1981). Unlike the physiological responses of an animal to ANF (such as increased pancreas weight and trypsin activity), an in vitro model remains static to these factors in terms of the amount and activity of enzymes (Pedersen and Boisen, 1982). This is reflected by greater influence observed on nutrient digestibility of fiber and ANF in in vivo compared to in vitro assays.

In in vitro models, dietary effects on passage rate and viscosity are not simulated (Weurding, 2002). Weurding et al. (2001) reported that diets comprising significant amounts of slowly digestible and resistant starch resulted in longer retention times in the small intestine of poultry. Moreover, in the in vivo situation, GIT capacity adapts to changes in the feeding program (Corring et al., 1989). If the diet contains a high protein level, pancreatic proteolytic enzymes will be increased, and an increase in lipid and starch contents will increase secretion of lipases and amylases, respectively. These characteristics of the GIT cannot be mimicked in vitro system (Boisen and Eggum, 1991). In addition, in the in vitro digestibility process, digestion products are not removed from the system. Ideally, the end-products should be continuously removed from the environment to avoid any feed-back effects and any surplus enzymatic activity in the in vitro environment (Boisen and Eggum, 1991).

Any strategy, including feed processing, with an effect on feed intake cannot be simulated in an in vitro assay. Feeding pelleted diets, through facilitating easy prehension, increases feed intake and reduces digesta retention time and consequently digestibility of major dietary nutrients (Abdollahi et al., 2011; 2013a,b). Negative effect of high feed intake on digestibility of protein and amino acids has also been shown in pigs (Liu et al., 2020). Therefore, for the accuracy of in vitro assays, the feed intake effect on nutrient digestion and energy utilization need to be considered. It seems very difficult, if not impossible, to mimic the load of feed (at a feed intake level that is close to ad libitum) in digestive tract of the birds in in vitro system.

Two other factors, which have not been considered in an in vitro assay are particle size reduction of feed (Svihus, 2011) that occurs in the gizzard and whole grain feeding. In recent years, use of whole grain feeding in poultry diets has attracted attention due to their effects on functionality of gizzard and gut development and health. Whole grain feeding leads to a lower pH of gizzard contents (Gabriel et al., 2003; Engberg et al., 2004; Singh et al., 2014) and increased gizzard size (Abdollahi et al., 2018) which may lead to increased pepsin activity in gizzard contents. Further studies are warranted to investigate whether it is possible to consider this complex matrix of conditions in in vitro assays or not.

In in vitro assays, it is generally assumed that all soluble material is absorbable, but in heat-treated proteins, certain small peptides are soluble in the in vitro method but not absorbed in vivo (Butts et al., 2012). Finally, in vitro digestibility values are usually expected to be higher than the in vivo values of the same set of samples. The main reasons for this difference are the absence of endogenous secretions and loss of nutrients in in vitro digestion models (Furuya et al., 1979; Boisen and Eggum, 1991).

Overall, all in vitro assays suffer from inadequacies and are not perfect. Given the limitations discussed above, it is not realistic to expect absolute agreement between in vitro and in vivo measurements, but this is not to say that in vitro assays of no value (Moughan, 1999). Although they cannot be used as a basis for practical feed formulations, the use of in vitro techniques is attractive to screen and relatively rank samples of a given material or different materials. Some in vitro assays appear to be satisfactory for the evaluation of select ingredients under defined conditions (Moughan and Rutherfurd, 2008). In vitro prediction accuracy can be increased by providing more information on contents of various anti-nutritional factors and dietary fiber content of feed ingredients (Moughan, 1999).

## 7 Conclusions and perspectives

There is a continuing demand from the poultry feed industry to explore and develop rapid methods capable of assessing the nutritional quality of raw materials on a real-time basis. In this context, better exploration of nutrient digestibility remains the prime focus. But, the use of animal models for routine feed evaluation is time-consuming and expensive and, require dedicated facilities and trained personnel with special skills. Moreover, in vivo studies are facing increasing criticism on ethical grounds and it is likely that this pressure become more intense in the future. Due to these logistic limitations, there is increasing interest in using rapid in vitro methods as part of feed evaluation programmes. In an in vitro assay, every effort should be made to simulate digestive processes in the GIT of poultry, as closely as possible. However, inherent biological properties of the ingredients which can affect the animal digestive tract will be lost in an in vitro assay. Although differences exist between in vitro and in vivo results, in vitro assays do seem to simulate gastric and small intestinal digestion of poultry. The 2-step in vitro digestion followed by collection of undigested materials by filtration or centrifugation is useful in poultry feed evaluation, but this approach needs further validation. The validation of the results from in vitro model with in vivo study is a key factor for the success of the technique. For the accurate prediction of nutrient digestibility, ingredient-specific in vitro digestibility techniques may be required rather than a single technique. It would appear that multiple regression equations, based on in vitro digestibility estimates and important chemical components, may prove expedient compared to simple regression equations.

## Author contributions

**Faegheh (Fifi) Zaefarian** conceived the idea for the manuscript and contributed to writing and editing of the paper and led the article. **Aaron Cowieson**, **Katrine Pontoppidan** and **M. Reza Abdollahi** critically revised the manuscript. **Velmurugu Ravindran** reviewed, added intellectual content and approved the final version of the manuscript to be published.

## Conflict of interest

We wish to confirm that there are no known conflicts of interest associated with this publication and there has been no significant financial support for this work that could have influenced its outcome.
